# Problem/case-based learning with competition introduced in severe infection education: an exploratory study

**DOI:** 10.1186/s40064-016-3532-3

**Published:** 2016-10-21

**Authors:** Jian-Hua Lei, Yi-Jing Guo, Zi Chen, Yao-Yan Qiu, Guo-Zhong Gong, Yan He

**Affiliations:** 1 Department of Infectious Diseases, The Second Xiangya Hospital, Central South University, No. 139 Middle Renmin Road, Changsha, 410011 Hunan People’s Republic of China; 2 Eight-Year Clinical Medicine, 2010 Grade, Xiangya School of Medicine, Central South University, No. 172 Tongzipo Road, Changsha, 410013 Hunan People’s Republic of China

**Keywords:** Problem/case-based learning, Competition mode, Clinical course, Severe infection

## Abstract

**Background:**

Problem/case-based learning (PCBL) is one of the most commonly used educational methods in medical schools.

**Aim:**

To further improve PCBL in clinical course of severe infection by introducing competition mode.

**Methods:**

Two classes of medical students were divided into two groups by class-based simple randomization and were taught the course of severe infection by PCBL. A team-based competition was introduced in the study group (n = 35) while not in the control group (n = 36). After the course, four closely associated references were recommended. All the students were notified about a group consultation on a similar case. In the final examination, a case with severe infection complicated with infectious shock was presented for the students to analyze and resolve listed questions. Their performances were qualitatively evaluated to justify the effectiveness of the competition-based PCBL.

**Results:**

The students in the study group were more active and initiative in case discussion and interaction, in referring to case-related articles and attending clinical group-consultation. They had better performance in the case analysis in the final examination. The typical case analysis test easily figured out more excellent students in the study group.

**Conclusions:**

The PCBL with competition mode introduced in is an effective approach to guide students to fully understand the clinical diagnoses and treatment of severe infection. It also prompts medical students’ initiative in referring to case-related articles and attending group-consultation, both of which are essential to equip medical students with sufficient competency for clinical practice.

## Background

Learning is a process which results in some changes or modifications in the learners’ ways of thinking, feeling and doing. The medical undergraduates mainly achieve their knowledge and skills through theoretical teaching and clinical practice. A very significant method in testing the teaching effect of medical education is to evaluate students’ basic ability including both the capability to make diagnoses and the practical ability to implement the treatment plans.

Medical courses used to be taught in traditional education approaches by means of tutorials, didactic lectures and practical classes. They are teacher-centered, with minimal active participation from the students, leading to lack of critical thinking in students and insufficient training targeted at integrating skills (Zahid et al. 2016).

Critical thinking is of great importance to physicians’ evolving clinical expertise (West et al. 2000). Its development needs an education system featured by a student-centered process. In this process, the teacher is hoped to use various innovative teaching methods to get the students motivated for meaningful learning rather than just passively receiving information, to get them actively participate in the process of learning and prepare themselves for a lifelong self-directed learning.

Problem-based learning (PBL) is a student-centered pedagogy in which students learn through solving listed questions. In PBL, students focus on complex problems without standard answers. They work in collaborative groups to identify what they need to learn in order to solve these problems (Tyler et al. 2009). The student is inculcated with capabilities to work productively as a team member, to master communication skills, to develop better clinical reasoning skills and presentation skills, to make decisions in unfamiliar situations, and to respect others, which are all key areas of a student’s education in community medicine (Schwartz et al. 1997; Khan and Fareed 2001). PBL encourages critical thinking, independent responsibility for learning, knowledge acquisition, sharing information, effective time management and better retention of information. It thus stimulates higher-order learning and helps achieve high professional competency (Wood 2008; Schwartz et al. 1997). Students in PBL classes have higher attendance and academic performance (Peters et al. 2000). The PBL approach to learning in medical education is the most significant educational innovation in the past four decades. Medical students learning by PBL approach obtained significantly higher knowledge and skill scores (Meo 2013), had increased learning and recalling output (Imanieh et al. 2014), excellent academic performance and higher success rates in examinations (Joseph et al. 2016), enhanced problem-solving skills and analytic skills (Shamsan and Syed 2009), as well as outstanding clinical reasoning skills (Tayyeb 2012). They were better at integrating basic science knowledge with clinical cases (Callis et al. 2010).

Case-based learning (CBL) is another popular student-centered teaching method. In the CBL, an authentic clinical case is given as a stimulus. The teacher is no longer a lecturer but a guider leading the process instead of giving the information directly. Exposing students to complex clinical cases promotes self-directed learning, clinical reasoning, clinical problem-solving and decision making. CBL generated the medical students’ learning enthusiasm, facilitated the health professionals’ deeper conceptual understanding, improved nursing students’ patient assessment skills and fostered more active and collaborative learners (Zhang et al. 2012; Thistlethwaite et al. 2012; Raurell-Torredà et al. 2015; Nordquist et al. 2012).

As a combination of CBL and PBL, problem/case-based learning (PCBL) has all of their advantages. PCBL can prompt students to develop team spirit in study and foster competitive learning mode and deep understanding of the knowledge relevant to teaching contents. PCBL has some other advantages including improving learning ability and other skills, encouraging self-assessment and logical thinking, integrating theory with practice, and developing students’ personalized learning by arousing internal and external enthusiasm (Aljarallah and Hassan 2015). Nowadays, PCBL is gradually becoming popular in medical education all over the world.

In spite of all these merits, doubts towards the effectiveness of PBCL existed (Carrero et al. 2007, 2008). So a new booster was needed. Based on the facts that team-based competition could increase resident physicians’ participation in quality-improvement education (Scales et al. 2016) and enhance weight loss outcomes (Leahey et al. 2012), a competition based PCBL teaching approach was tried.

In the selection of the teaching cases in PCBL, we focused on cases with severe infections, for severe infection is a difficult chapter in the clinical courses of infectious diseases for teaching. In the past, teaching of the infectious diseases almost all followed the hints of etiological and epidemiological characteristics, clinical manifestations, laboratory tests, diagnosis and differential diagnosis, treatment and prevention of the diseases. Selection of the representative clinical cases in the CBL courses and guiding question lists in the PBL courses both followed the same regime. However, the pathogens leading to severe infections are generally unidentified, so in practice, judgment on the progress and treatment of a disease are taught from the perspectives of changes of the patient’s condition and its underlying pathophysiological mechanisms. That is, it can’t be taught in the same approach for a traditional infectious disease with a definite pathogen. A case-based and problem-driven teaching approach is essential to cultivate and mobilize clinical thinking of the medical student and to help them to develop a broader perspective of case scenarios.

Based on the above considerations, we introduced competition into PCBL in the teaching of severe infection to hope for better teaching effects. This study was hence done to compare the academic and clinical performance of students taught severe infections by competition based-PCBL methodology with that of students by regular PCBL method.

## Methods

### Curriculum

Infectious Diseases published in 2010 by the People’s Medical Publishing House of People’s Republic of China was used as the textbook. And the multimedia teaching courseware was made by the same crew of the Department of Infectious Diseases, the Second Xiangya Hospital, Central South University. The courses were taken in a classroom and lasted for 2 h.

### Teaching subjects and management

We recruited seventy-one students from two classes of the same major who were studying clinical courses and on probation in the Department of Infectious Diseases, the Second Xiangya Hospital, Central South University. They all had completed the 3-year education on basic medicine and half a year-basic education of clinical skills. The students were divided into two groups by class-based simple randomization. The ratio of males to females was balanced between the two groups. All of the students were taught the same course of severe infection by PCBL. The students in the study group (n = 35) were divided into five teams and taught by competition-introduced PCBL. The students in the control group (n = 36) were taught in a regular PCBL way. The teaching approaches for the other chapters of infectious diseases and the related probation practice were the same for both groups.

### Teaching case and listed questions

**Fig. 1:**
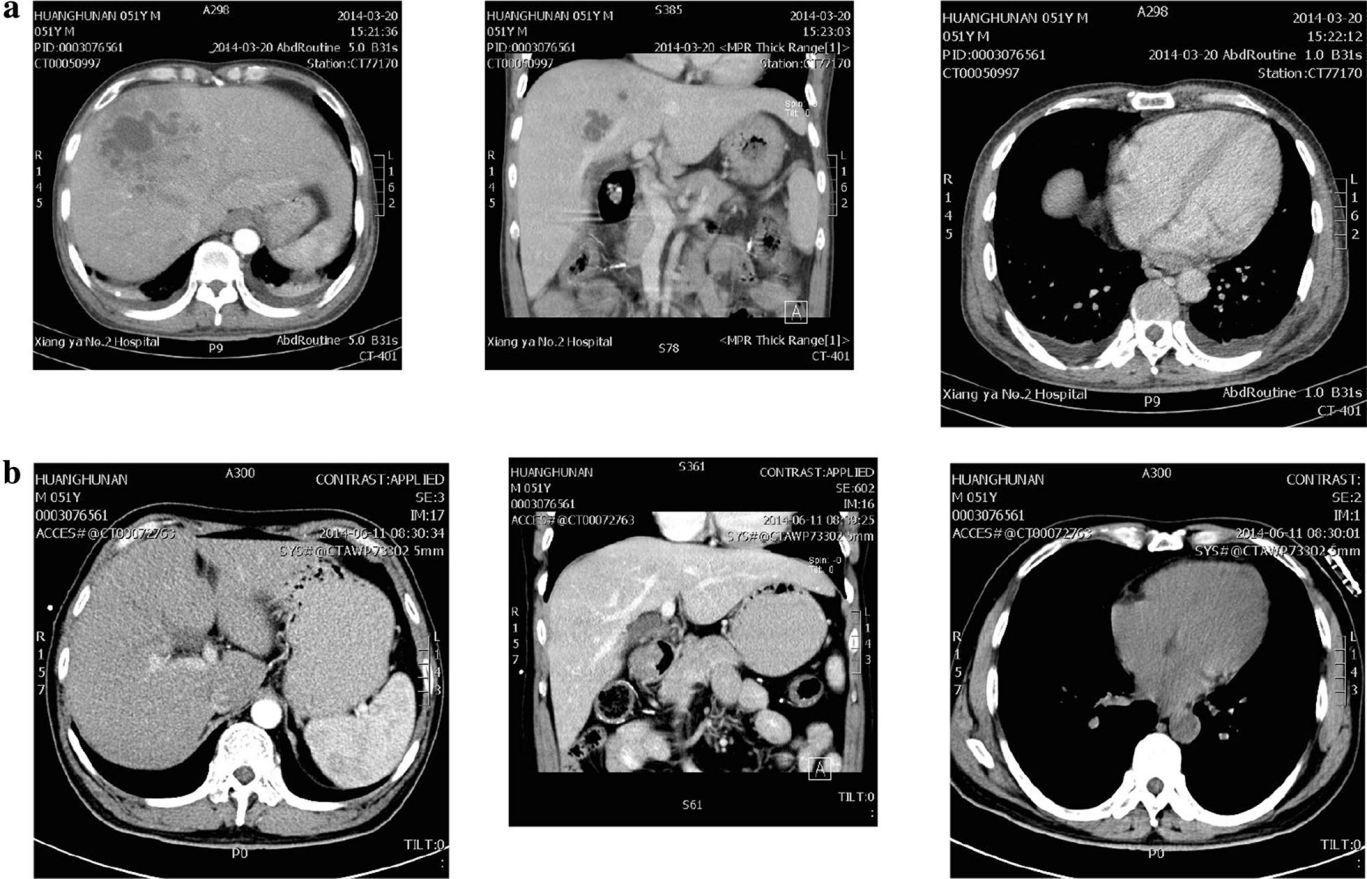
Computed tomography (CT) examination results of the teaching case. **a** Before CT-guided percutaneous aspiration and catheter drainage in the management of liver abscesses. **b** Three months after CT-guided percutaneous aspiration and catheter drainage in the management of liver abscesses

The medical history of the case used in the teaching course was as follows. A middle-aged male had diabetes for several years and did not have a good control of blood glucose. A community general physician treated him inadequately when he suffered from a slight infection in late February, 2014. Then the infection developed into a severe *Klebsiella pneumoniae* sepsis with multiple organ dysfunction syndrome (MODS), infectious shock and liver migrating abscesses (see Fig. 1). After correct clinical and etiological diagnoses in our hospital, the patient received proper treatment and finally recovered.

The 16 guiding questions for the PCBL teaching were listed below. They were about the incentive, etiology, pathogenesis, clinical and etiological diagnoses and prognosis of severe infection and principles for treatment of infections, complications and infectious shock.

(A) What are the clinical features of a fever caused by an infection?
(B) Are the treatment measures by the community general physician reasonable?
(C) What are the common causes of severe infections?
(D) If you had received the patient when he was transferred to your hospital, what kinds of first aid treatment would you prescribe?
(E) The patients had clinical manifestations of a shock. Please describe the clinical classifications of shock according to its etiology. What kind of shock was the patient complicated with?
(F) What are the common pathogens associated with infectious shock?
(G) Epidemic hemorrhagic fever is endemic in Hunan Province in later February. So what clinical hints conduce to excluding the probability of epidemic hemorrhagic fever in this case?
(H) What are the molecular mechanisms of infectious shock induced by severe infections?
(I) Could you list some up-to-date biomarkers conducive to the early diagnosis of a severe infection and the subsequent infectious shock?
(J) Please describe the hemodynamic characteristics of severe infections and subsequent infectious shock.
(K) Why metabolic acidosis and hyponatremia often occur in patients suffering from infectious shock?
(L) Please list the treatment principles of infectious shock according to its hemodynamic characteristics and analyze the principles of early fluid resuscitation and application of vasoactive agents.
(M) Many patients have stress hyperglycemia in infectious shock. A great variety of clinical researches propose sustained monitoring and control of the blood glucose of the patients with severe infections. When multiple tests find increased blood glucose levels and strongly positive results in uric glucose tests, how to identify their origin from stress hyperglycemia or from diabetes?
(N) Opportunistic infections are common in diabetic patients. What is the mechanism of immunocompromise in them?
(O) Many retrospective clinical researchers have found that for the diabetic patients complicated with *Klebsiella pneumoniae* sepsis, especially when there are obvious lung infection foci, there is a great possibility of missed diagnosis of liver abscess. Could you delineate the possible reasons and preventive measures from the view of a clinical doctor?
(P) Please list several main mechanisms of drug resistance of resistant *Klebsiella pneumoniae*.

### Competition mode

The study group was divided into five teams. The listed 16 case-relevant questions were classified into two categories, required questions and quick response questions. Each team answered the required questions in turn. When one team answered the required questions, their performance was marked by the other teams and teachers. Before answering questions or evaluating answers, the teams were given 5 min for full discussion among team members. Interactive behavior like summarizing, challenging and evaluating was encouraged within confined time. All of the teams were given adequate time to answer questions or rate other teams’ performance. In the end, one team was judged to be the winner and awarded in view of the integrity and accuracy of their answers as well as the objectivity and equity of their scoring practice. Two cinema tickets were provided for each winner as an award.

### Teaching efficiency assessment

The teaching efficiency was assessed from four aspects as listed in Table 1.

**Table 1:** Assessment of teaching efficiency

Item	Assessment methods
Participation in discussion and interaction	The number of the students participating in discussion and interaction for each question was recorded and their participation degree was described as participation person-times. For example, for Question 1, 8 students participate in discussion, 3 answer questions and 4 challenge others’ answers, then the group’s participation in Question 1 was 15 person-times. The participation person-times for each question are summed up to get the overall person-times for the teaching course
Initiative in referring to articles	After the course, a list of four referable articles closely associated with the case, including international guidelines for management of severe sepsis and septic shock composed by the Surviving Sepsis Campaign Guidelines Committee, were provided to the students in both groups. Two weeks after the course, the number of the students referring to the recommended articles and the number of the articles referred to by each student were investigated
Initiative in participating in a group consultation	Within 2 weeks after the course, all the students were notified that a group consultation on a similar case with severe infection would be held in the infection wards, and they could participate in it on their own initiative. The proportion of the students participating in the consultation in each group was calculated
Performance in a case analysis in the final examination	At the end of the semester, the participants were assigned a task in the final examination without prior notice to analyze a case with severe infection and infectious shock, to make a diagnosis and a clinical treatment scheme. The scoring rates of correct diagnoses and proper clinical treatments were calculated. For example, 10 key points were delineated for a full answer for the correct diagnosis, and the students listed 7 points, then the scoring rate of the student for the diagnosis was expressed as 0.7. Besides, the discrimination value in this case analysis was analyzed. All the indicators listed above were compared between the study group and control group for evaluating the teaching effectiveness

### Statistical analysis

All statistical analyses were performed with IBM^®^ SPSS^®^ Statistics version 20.0, using descriptive statistical indexes such as rate, ratio, mean and standard deviation (SD), et al. Chi squared test was performed for comparison of rates and ratios. Analysis of Variance (ANOVA) and Kruskal–Wallis H test were performed for comparison of means. One sample Kolmogorov–Smirnov test was used to verify the normal distribution of data sets. For all these tests, *P* value less than 0.05 was considered statistically significant.

## Results

### Participation in discussion and interaction in the course

The person-times and constitutions of the students participating in discussion, answering questions initiatively and further challenging or analyzing others’ answers were all significantly higher in the study group than in the control group (see Table 2). The overall person-times and constitutions of the students participating in interaction actively were also statistically different (χ^2^ = 29.762, *P* = 0.000).

**Table 2:** Comparison of the person-times of participation in discussion and interaction for each question and the total between two groups

Question	Study group (n = 35)				Control group (n = 36)				*P* value
	Participating in discussion	Answering questions initiatively	Further challenging or analyzing others’ answers	Total	Participating in discussion	Answering questions initiatively	Further challenging or analyzing others’ answers	Total	
1	8	3	4	15	2	1	3	6	0.001^a^
2	7	2	5	14	3	2	2	7	0.094
3	4	2	4	10	2	3	4	9	0.761
4	5	1	5	11	3	3	2	8	0.432
5	6	1	6	13	4	1	3	8	0.223
6	8	3	3	14	2	4	4	10	0.347
7	5	3	3	11	5	4	2	11	0.944
8	7	2	3	12	2	2	3	7	0.205
9	5	3	4	12	0	2	1	3	0.014
10	5	3	2	10	0	3	1	4	0.087
11	7	1	5	13	3	2	2	7	0.140
12	4	2	3	9	2	2	1	5	0.246
13	5	3	2	10	3	3	1	7	0.413
14	7	2	5	14	4	1	2	7	0.094
15	6	2	3	11	2	1	1	4	0.053
16	8	1	4	13	2	2	1	5	0.042
Total				192				108	0.000^b^

Unit: person-time

Supposed total person-times for each question for each group = 3 × n

Supposed total person-times for all the questions for each group = 3 × 16 × n

^a^Comparison between 15/(3*35) for the study group and 6/(3*36) for the control group

^b^Comparison between 192/(3*16*35) for the study group and 108/(3*16*36) for the control group

### Performance in referring to the references provided by teachers

Totally, 51.4 % (18/35) of the students in the study group referred to the references provided by the teachers on their own initiative, and the proportion was significantly higher than that in the control group (13.9 %, 5/36, χ^2^ = 11.419, *P* = 0.001). The average pieces of articles read by the students initiatively in the study group were also significantly higher (0.85 ± 0.97 vs. 0.25 ± 0.69, Kruskal–Wallis H test, χ^2^ = 10.431, *P* = 0.001).

### Attendance in group consultation

Totally, 25.7 % (9/35) of the students in the study group attended the group consultation initiatively, while only 5.6 % (2/36) did in the control group, with statistically significant difference (χ^2^ = 4.076, P = 0.043).

### Performance in the clinical case analysis in the final examination

The rates of correct diagnoses and of the correct answers to the listed questions about key points in clinical treatment of infectious diseases in the final examination were compared between the two groups.

The scoring rate in the case analysis was neither high in the study group (0.62) nor in the control group (0.51), which conformed to the characteristics of the medical students who didn’t enter the internship phase. The scores of the students in the study group who had the competition mode were significantly higher (P < 0.05). So were the rates of correct diagnosis (74.3 vs 58.3 %) and correct answer to the critical points in clinical handling of patients with severe infection (65.6 vs 44.8 %) (P < 0.01) (see Table 3).

**Table 3:** Comparison of the rates of correct diagnoses and of correct answers to listed questions about key points in clinical treatment of infectious diseases in the final examination between two groups

Parameters	Study group (n = 35)	Control group (n = 36)
Average scores	9.26 ± 2.97*	7.65 ± 2.48
Number (rates) of students with correct diagnoses (n, %)	26 (74.3)*	21 (58.3)
Rates of correct answers to the listed questions (%)	65.6*	44.8

* P < 0.05

## Discrimination value of the case analysis test in the final examination

For the students in the study group who were taught by the competition mode, the scoring rates of the case analysis in the students with total score ranking within the top 27 % and within the last 27 % in the infectious disease final examination were 86.3 and 50.0 % respectively, and the discrimination value was 0.36. As for the students in the control group without competition mode, the scoring rates of the case analysis in the students with total score ranking within the top 27 % and within the last 27 % in the infectious disease final examination were 65.3 and 43.3 % respectively, and the discrimination value was 0.22 (see Table 4). It indicated that such typical case analysis was more suitable for testing the clinical competence of the students actively participating in interaction learning.

**Table 4:** Discrimination value for excellent students in the infectious disease final examination by a case analysis test

Parameters	Study group (n = 35)	Control group (n = 36)
Average points of last students	7.5	6.5
Average points of top students	12.9	9.8
Discrimination value	0.36	0.22

## Discussion

Medical graduates today are facing numerous emerging diseases and are particularly expected to be critical thinkers and self-directed learners. They are supposed to have generic skills like effective communication and teamwork besides problem-solving ability in activities including making disease diagnosis, formulating treatment strategies and investigating epidemics.

World Health Organization (WHO) consultations on public health teaching and training recommend student-centered, inquiry-driven, problem-oriented and evidence-based innovative learning methods in public health courses. The teacher was expected to teach students by student-centric and problem-based approaches, playing a role as a facilitator to help students to acquire these competencies (WHO 2010).

PCBL, a teaching mode innovation totally different from one-way indoctrination classes in the traditional teaching, meet the needs.

In our study, a further step was taken. A competition mode with two cinema tickets as an award was introduced into the PCBL, which did improve the teaching efficacy. Despite the motivation by the award, the winners were more moved by the sense of achievements. The students taught by competition—based PCBL approach devoted themselves more in the discussion and interaction in the course. They were more active and initiative in referring to the case-related articles and in participating in the clinical group-consultation. Both practices were essential to cultivate the students’ comprehensive ability to cope with diagnosis and treatment of complicated cases, and also conducive to helping them to develop a habit to effectively integrate research articles with clinical practice. In the final examination, these students had a better performance. They had a higher rate of correct diagnoses as well as higher scores than the controls. Competition introduced PCBL could motivate the students to think more actively and deeply and fostered more excellent students than the regular PCBL.

Sense of self-respect and desire for winning are characteristics of the students at college age, which enables the competition mode as an important promoter. Besides, dividing the group into several teams could initiate more communications and inspirations. The competition mode inspired students’ interest and enabled them to focus on the cases and listed questions and to recall and integrate related knowledge to interpret the cases and solve the problems. The practice of comparing the answers of students with teachers emphasized the significance of clinical experience and practice. Moreover, different forms of competition gave everyone the chance to demonstrate their competency. Despite some critical overview on the effectiveness of PBL and CBL in medical education (Al-Azri and Ratnapalan 2014; Chilkoti et al. 2014), students preferred to problem-based learning over lecture-based learning because of motivation boost, knowledge retention, class attractiveness and practical use (Joseph et al. 2016).

Based on the results of our study, PCBL with competition mode introduced in medical teaching conduces to development of clinical reasoning, critical thinking and self-directed learning skills and helps in developing a broader perspective of case scenarios. It is proposed to be applied in the teaching of medical science. Popular use of Electronic Healthcare Record (EHR) in hospitals provided a large database for selection of real patient stories as educative cases Ricci et al. 2016).

As for colleges and universities other than medical ones, to maximize the effectiveness of PBL, PBL curricula should be revised according to their own needs. Taking the characteristics of different subjects into full consideration, better alignment between PCBL and the reigning teaching and learning regime, frequent check the weaknesses of the implementation process and promotion of the future use of the checklist are key to successful implementation of PCBL in medical undergraduate curriculum.

## Conclusions

The PCBL with competition mode introduced in is an effective approach to guide medical students to fully understand the clinical diagnoses and treatment of severe infection. It also prompts medical students to initiatively and consciously refer to case-related articles and participate in related group-consultation, both of which are essential to equip medical students with competency sufficient to face clinical practice.

## Abbreviations

PBL: problem-based learning
CBL: case-based learning
PCBL: problem/case-based learning
WHO: World Health Organization
MODS: multiple organ dysfunction syndrome
ANOVA: analysis of variance
EHR: electronic healthcare record
CT: computed tomography
